# Supplementary material for: The DEAD-Box RNA Helicase Ded1 Is Associated with Translating Ribosomes
Source: Genes (Basel). 2023 Jul 31;14(8):1566. doi: 10.3390/genes14081566 (PMC10454743; doi:10.3390/genes14081566)
Supplement: Supplementary file 1 [file genes-14-01566-s001.zip › genes-2500344-supplementary/genes-2500344--Yeter-Alat_Supplementary Data.pdf]

Supplementary Data: The DEAD-Box RNA Helicase Ded1 Is Associated with Translating Ribosomes

Hilal Yeter-Alat, Naïma Belgareh-Touzé, Emmeline Huvelle, Josette Banroques and N. Kyle Tanner

SUPPLEMENTARY MATERIAL AND METHODS

Protein characterization for Supplementary Figure S1B.

Approximately 1.9 ml of crosslinked cells were recovered by centrifugation at maximum speed and the total proteins were rapidly extracted as previously described with minor modification [1]. The pellets were resuspended in 150 µl 1.85 N NaOH with 7.4% β-mercaptoethanol and incubated on ice for 10 min. Then 150 µl of 50% TCA was added to the samples, and they were incubated 10 min on ice. Proteins were pelleted at 13,200 rpm in a Eppendorf 5415R centrifuge at 4°C, washed twice with ice-cold 100% acetone and air dried. The samples were resuspended in SDS loading buffer and the proteins electrophorectically separated on a 10% SDS-PAGE. The proteins were subsequently electrophoretically transferred to nitrocellulose membranes (Amersham Protran) that were probed with IgG specific for Ded1 and PGK1. The blots were resolved with a Clarity Western ECL chemiluminescence kit (Bio-Rad) and visualized with a ChemiDoc XRS+ and Image Lab software (Bio-Rad)

Protein-RNA characterization for Supplementary Figure S2B.

Crosslinked protein-RNA complexes on the IgG-Protein A-Sepharose beads were digested with nuclease S1 and then directly eluted with 0.1 M glycine, pH 2.3, as described above (no adapter ligation step). The pH was adjusted to ~7 with NaOH, the solution was made 150 µg/ml, final, in sodium deoxycholate and then the proteins were precipitated with 15% TCA for 30 min on ice. The precipitated proteins were recovered by centrifugation at 13,200 rpm speed in an Eppendorf 5415R, the pellet washed twice with cold acetone, and then dried. The proteins were resuspended in SDS loading buffer, electrophorectically separated on a 10% SDS-PAGE, transferred to nitrocellulose membranes and then probed with Ded1-specific IgG.

3' end-labeling of the RNA for Supplementary Figure S2C.

The optimization of the crosslinking conditions and the recovery of the RNA was monitored by 3' ^{32}P-pCp end-labeling of the RNA. After crosslinking, binding on IgG-Protein A Sepharose beads and S1 digestion, the protein-RNA complex was eluted with 0.1 M glycine, pH 2.3, as described above, and the pH adjust to ~7 with NaOH. The proteins in the solution were degraded by the addition of proteinase K and extracted with an equal volume of phenol. The aqueous layer was subsequently extracted with an equal volume of chloroform-isoamyl

alcohol (24:1), and then the RNA was precipitated by the addition of 2.5 volumes of ethanol and by placing at -20°C overnight. The RNAs were recovered by centrifugation at 13,200 rpm for 20 min at 4°C in an Eppendorf 5415R, washed with 400 µl cold 70% ethanol and dried. The pellets were dissolved and the RNAs labeled in 150 µl volumes with 10 units of T4 RNA ligase (New England Biolabs) in the provided reaction buffer with 250 µCi of ^{32}P-pCp (3000 Ci/mmole; American Radiolabeled Chemicals Inc) overnight at 4°C. An equal volume of 10 M urea loading buffer was added that contained 20% sucrose, 0.02% xylene cyanol, 0.02% bromophenol blue, 0.5% SDS, 10 mM Tris-base, 8.3 mM boric acid and 0.1 mM EDTA, and the RNAs were electrophoretically separated on a 6% polyacrylamide gel containing 7 M urea. The images were visualized with a Typhoon FLA9500 phosphoimager (GE Healthcare) using a BAS MS imaging plate (Fujifilm).

Supplementary Table S1. Outline and comparison of PAR-CLIP techniques

Individual steps	Classic PAR-CLIP [a]	qtPAR-CLIP
4-thiouracil	100 µM for 18h	300 µM for 5h
Irradiation	365 nm (no lid)	365 nm (with polystyrene lid)
Lysate RNase digestion	Partial RNase T1 digestion of Lysate (1 U/µl)	No. Less complex recovered (see Supplementary Fig. 2)
IgG-protein-A/G beads	Magnetic-G; bind protein in lysate	Sepharose-A; bind protein in lysate
Nuclease digestion of bound complexes	Second RNase T1 digestion (100 U/µl)	Single nuclease S1 digestion (0.5 U/µl)
Dephosphorylation and 5' labelling of crosslinked RNA	RNA 3' dephosphorylated and 5' labeled using T4 PNK and [γ-^{32}P-ATP]	No. Nuclease S1 leaves a 3' hydroxyl. Dephosphorylation unnecessary
Elution of complexes	Yes	No
Purification of protein RNA-complexes	RNA-protein complexes SDS-PAGE purified	No. Stringent washing of the IgG-bound protein-RNA; no loss due to gel extraction[b]
On-bead 3' adapter ligation	No	3' adapter added; washing removes free adapter; no adapter concatemers
Elution of complexes	No (previous step)	Yes
Protease K treatment	Yes	Yes
RNA extraction	Yes	Yes
3' adapter ligation & gel purification	Yes	No (added on beads)
5' adapter ligation	Yes	Yes
PAGE Gel purification	Yes	No
RT-PCR	Yes	Yes
Gel purification of cDNA	Yes	Yes
Total steps	15	11
Duration	6 days	≤ 4 days

[a] Based on protocol of Hafner et al, 2010 [2]; also see [3].

[b] The wash buffer contained 1 M urea and 0.4 mg/ml heparin to reduce nonspecific RNA interactions, and it contained 0.1% Triton-X100 and 0.4 mg/ml BSA to reduce nonspecific protein interactions.

Supplementary Table S2. Oligonucleotides used in this study

Name [a]	Sequence
3’ adapter	5' PO_4-NNN NTG GAA TTC TCG GGT GCC AAG G-A[23ddC] 3' [b]
5’ adapter	5' AmC6-GT TCA GAG TTC TAC AGT CCG ACG ATC NNN rU-OH 3’ [c]
RT primer	5' OH-GCC TTG GCA CCC GAG AAT TCC A-OH 3'
RPI1	5’ OH-CAA GCA GAA GAC GGC ATA CGA GAT CGT GAT GTG ACT GGA GTT CCT TGG CAC CCG AGA ATT CCA-OH 3’
RPI2	5’ OH-CAA GCA GAA GAC GGC ATA CGA GAT ACA TCG GTG ACT GGA GTT CCT TGG CAC CCG AGA ATT CCA-OH 3’
RPI4	5’ OH-CAA GCA GAA GAC GGC ATA CGA GAT TGG TCA GTG ACT GGA GTT CCT TGG CAC CCG AGA ATT CCA-OH 3’
RPI9	5’ OH-CAA GCA GAA GAC GGC ATA CGA GAT CTG ATC GTG ACT GGA GTT CCT TGG CAC CCG AGA ATT CCA-OH 3’
RPI10	5’ OH-CAA GCA GAA GAC GGC ATA CGA GAT AAG CTA GTG ACT GGA GTT CCT TGG CAC CCG AGA ATT CCA-OH 3’
RPI12	5’ OH-CAA GCA GAA GAC GGC ATA CGA GAT TAC AAG GTG ACT GGA GTT CCT TGG CAC CCG AGA ATT CCA-OH 3’
FP1	5’ OH-AAT GAT ACG GCG ACC ACC GAG ATC TAC ACG TTC AGA GTT CTA CAG TCC GA-OH 3’
GFP/mCh 5'	GCC TAT **CTC GAG** GGA GCA GGT GCT GGT [d]
GFP/mCh 3'	GCC TAT **GTC GAC TTA CTT** GTA CAG CTC GTC CA [d]

[a] RT, reverse transcriptase; RP, reverse primer; FP, forward primer. RPI, 3' reverse PCR index primer, where the index of 6 nucleotides is underlined; FP1, 5' forward PCR primer; GFP/mCh 5', GFP/mCherry+XhoI-ed; GFP/mCh 3', GFP/mCherry_SalI-ed.
[b] 3’ end blocked with 2',3'-dideoxycytidine (23ddC); N is a random nucleotide
[c] 5’ end blocked with 5’ amino$(CH_2)_6$ (AmC6) ; N is a random nucleotide
[d] Regions of complementarity are underlined and restriction sites are shown in bold.

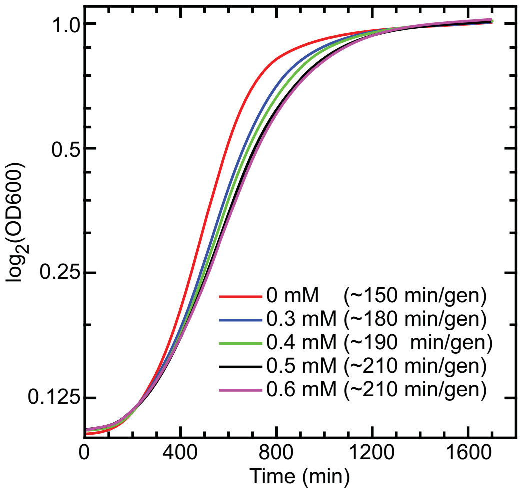

Supplementary Figure S1. The 4-thiouracil has little affect on cell growth.
Growth curves of the wildtype BY4742 *S. cerevisiae* strain cultured with five different concentrations of 4-thiouracil, from 0 to 600 μM, in SD-LEU medium incubated at 30°C. Two independent experiments were made for each 4-thiouracil concentration (n=2). The means for each condition are shown; the standard deviations were deleted for clarity. The generation times are as shown.

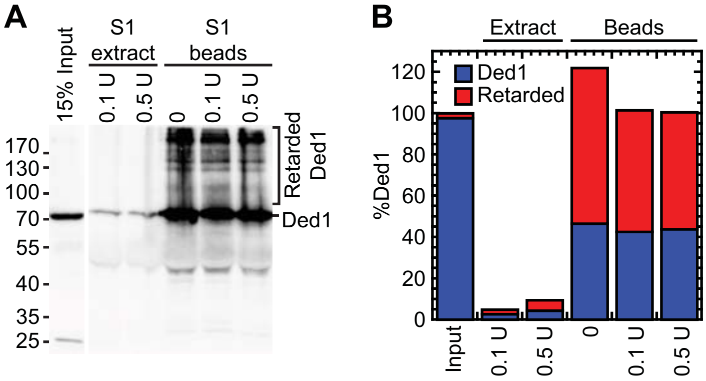

Supplementary Figure S2. Effect of S1 nuclease treatment before and during IP.
A, Western blot analysis of Ded1-RNA complexes isolated with Ded1-specific IgG bound on Protein A-Sepharose beads that were treated with the indicated concentrations of nuclease S1 either before (extract) or after (beads) binding Ded1 on the IgG-Protein A-Sepharose beads. The reduced recovery in the S1 extract was due to proteases in the extract, and probably due to the activation of zinc-dependent metalloproteases that are not affected by the added protease inhibitors [4]. The eluted RNA-Ded1 complexes were electrophoretically separated on a 10% SDS-PAGE, transferred to nitrocellulose membranes, and revealed with IgG specific for Ded1. **B**, Quantification of bands shown in (**A**). Values were normalized relative to the input value. All the chemoluminescence signal of material migrating above the free Ded1 was counted as retarded.

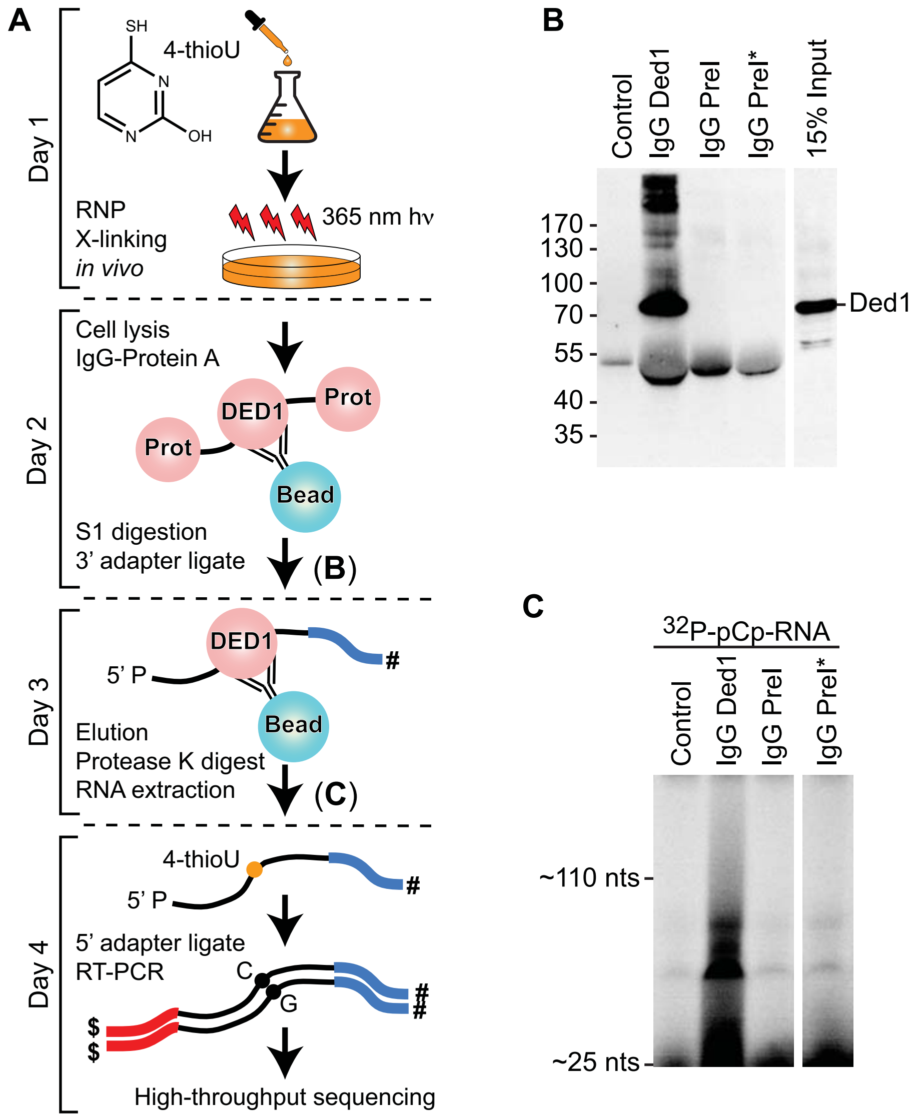

Supplementary Figure S3. Ded1-RNA interactions *in vivo* with the qtPAR-CLIP technique. A, Schematic representation of the qtPAR-CLIP technique. **B,** Western blot of PAGE-separated, crosslinked, RNA-Ded1 complexes isolated by immunoprecipitation (IP) with Ded1-specific antibodies (IgG Ded1). The control fractions used different pre-immune serums (IgG PreI; IgG-PreI*) or no antibody (Control). **C,** The eluted RNA-Ded1 complexes were protease K-digested, 3' ^{32}P-pCp-labeled and PAGE separated. For experimental details, see the Material and Methods section.

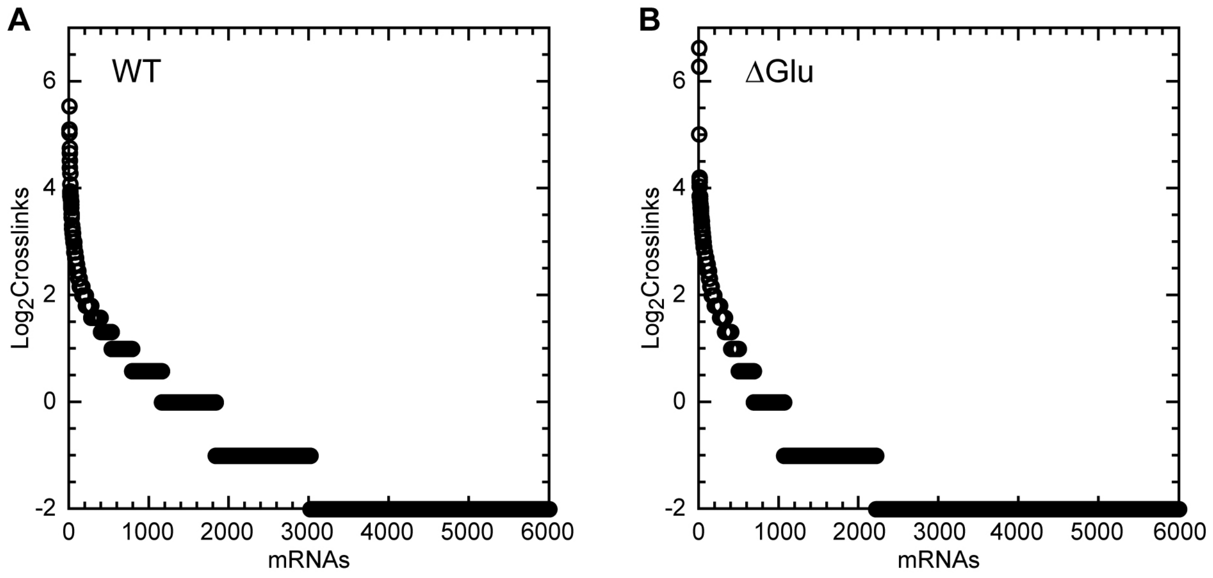

Supplementary Figure S4. Relatively few mRNAs were highly crosslinked to Ded1. The mean number of crosslinks are shown and those with no crosslinks were arbitrarily assigned a value of -2. **A**, Crosslinks resulting from cells growing under standard conditions (WT). **B**, Crosslinks resulting from cells grown for 15 min under glucose-depletion conditions (ΔGlu).

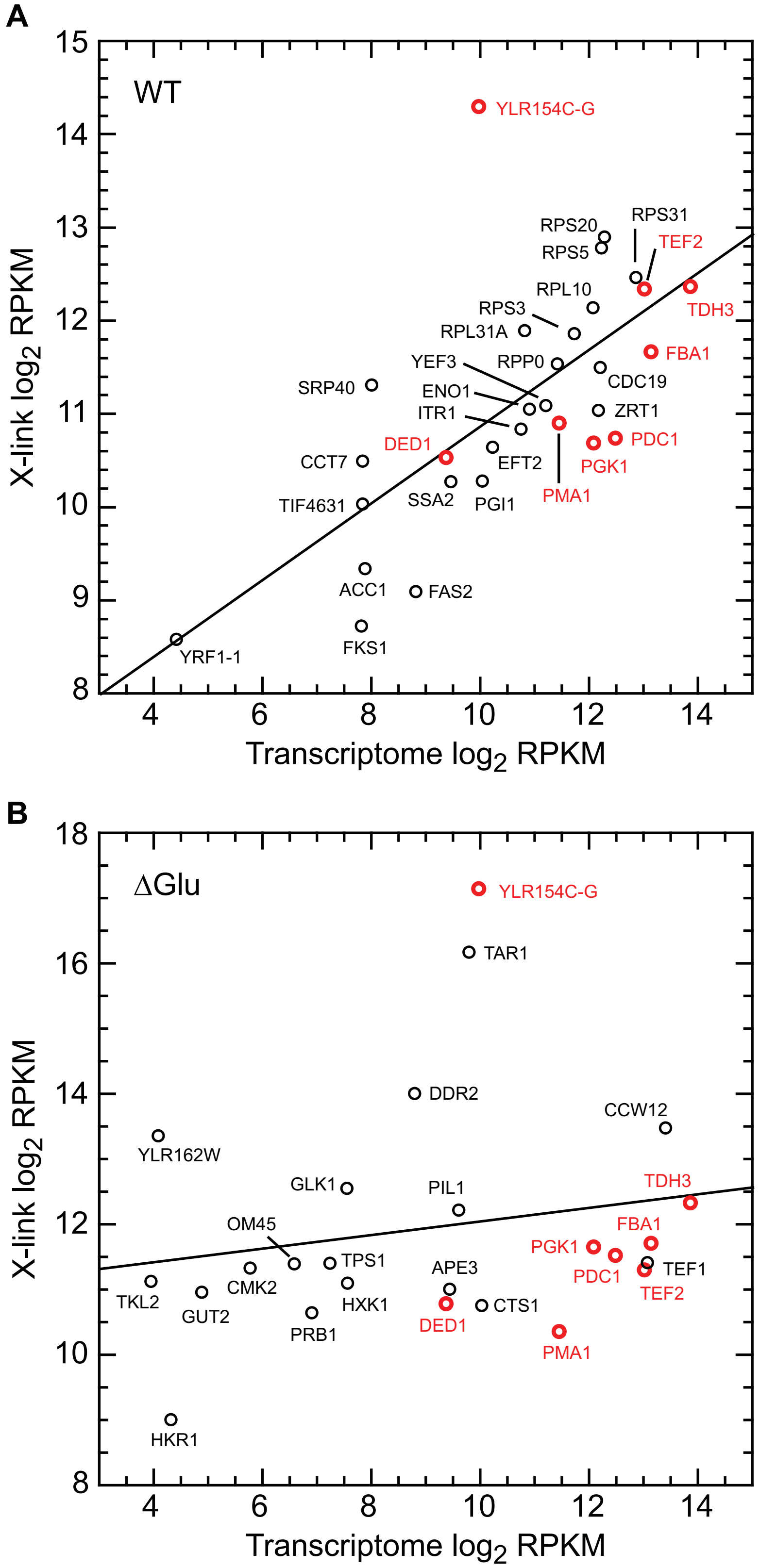

Supplementary Figure S5. The most crosslinked mRNAs. A, Crosslinked mRNAs from cells grown under standard conditions (WT). The linear regression slope is 0.412 with an R^2 of 0.473. **B**, Crosslinked mRNAs from cells grown under glucose-depletion conditions (ΔGlu). The linear regression slope is 0.104 with an R^2 of 0.035. Genes found under both conditions are shown in red.

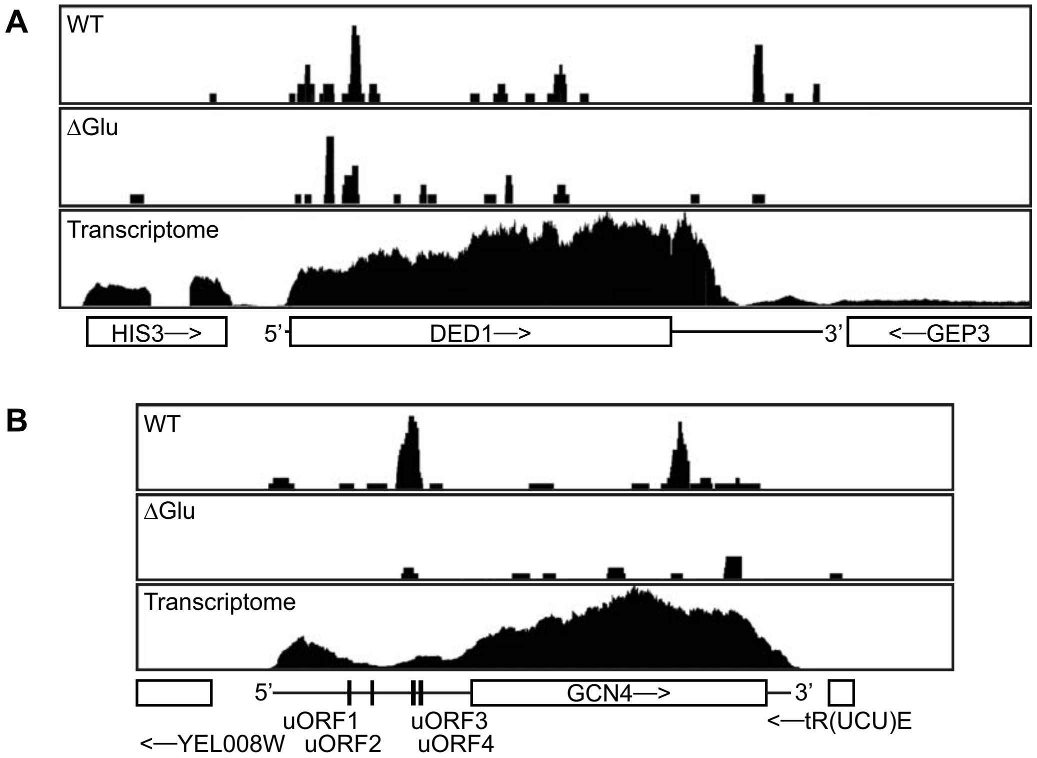

Supplementary Figure S6. Crosslinks occur at discrete sites on the mRNAs. A, Ded1 has a short 5' UTR of 21 residues, an ORF of 1814 and a 3' UTR of ~722. The Y-axis scale is 10 for the standard (WT) and glucose-depletion (ΔGlu) conditions and 2800 for the transcriptome. **B**, GCN4 has a long 5' UTR with four upstream ORFs (uORF) that regulate the expression of GCN4. The Y-axis scale is 15 for the standard and glucose-depletion conditions and 7100 for the transcriptome. Data were analyzed with Integrated Genome Browser (IGB) and visualized with the Integrated Genome Viewer (IGV; [5]).

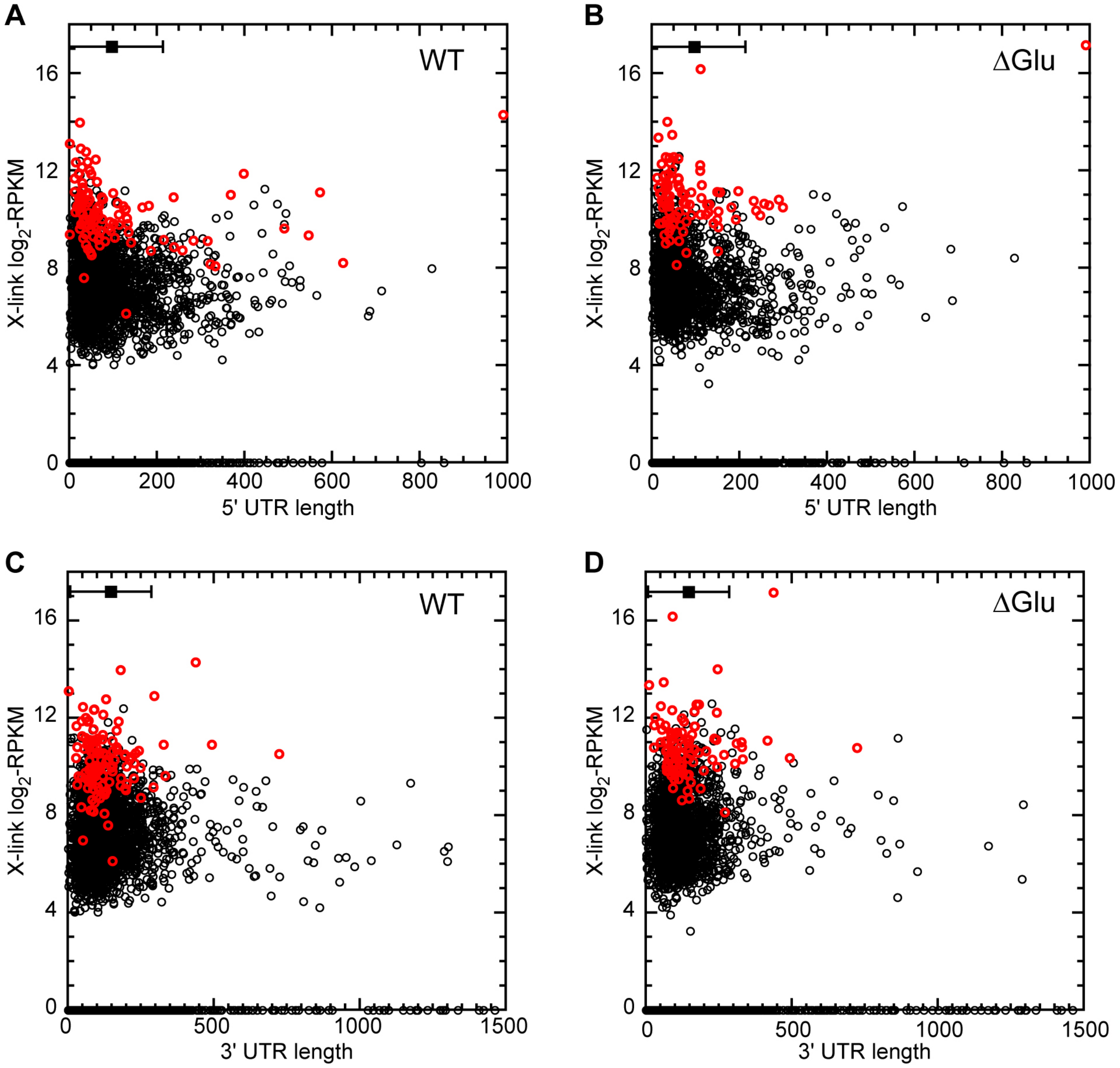

Supplementary Figure S7. Crosslinks were poorly correlated with UTR length. The RPKM of the crosslinked mRNA was plotted against the 5' and 3' UTR lengths for the mRNAs that were previously determined (6,7). The mean (■) and standard deviations of these lengths is as shown in the upper left corners. The mRNAs without crosslinks are arbitrarily given a value of 0. The mRNAs with a high frequency of crosslinks fragments are shown as red. Note that the lengths of the 3' UTRs on any particular mRNA can vary depending on the site of poly(A) addition [8]. **A** & **C**, Crosslinks on mRNAs for cells grown under standard conditions (WT) for the 5' and 3' UTRs, respectively. **B** & **D**, Crosslinks on mRNAs for cells grown under glucose-depletion conditions (ΔGlu) for the 5' and 3' UTRs, respectively

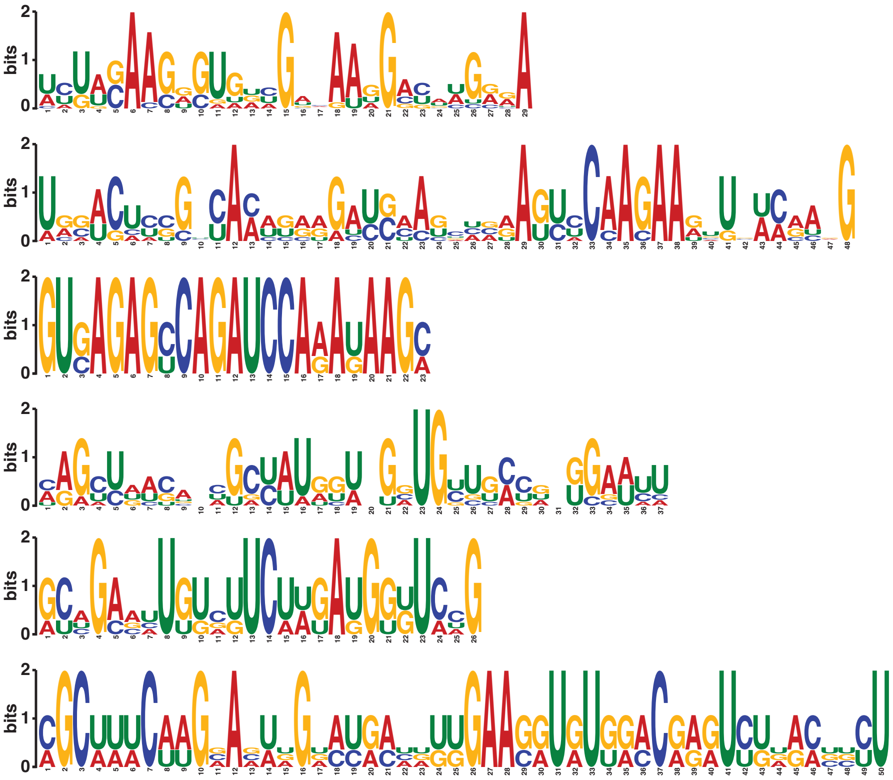

E-value	Sites	Width
4.9e-12	12	29
6.8e-7	6	48
9.7e-2	3	23
6.6e-1	7	37
5.4e-1	4	26
1.6e-2	3	50

Supplementary Figure S8. MEME Logo Sequence Motifs. RNA fragments with unique peak reads of 20 or more were analyzed with the MEME software and the Any Number of Repeat (ANR) default settings (https://meme-suite.org/meme/tools/meme; [9,10]).

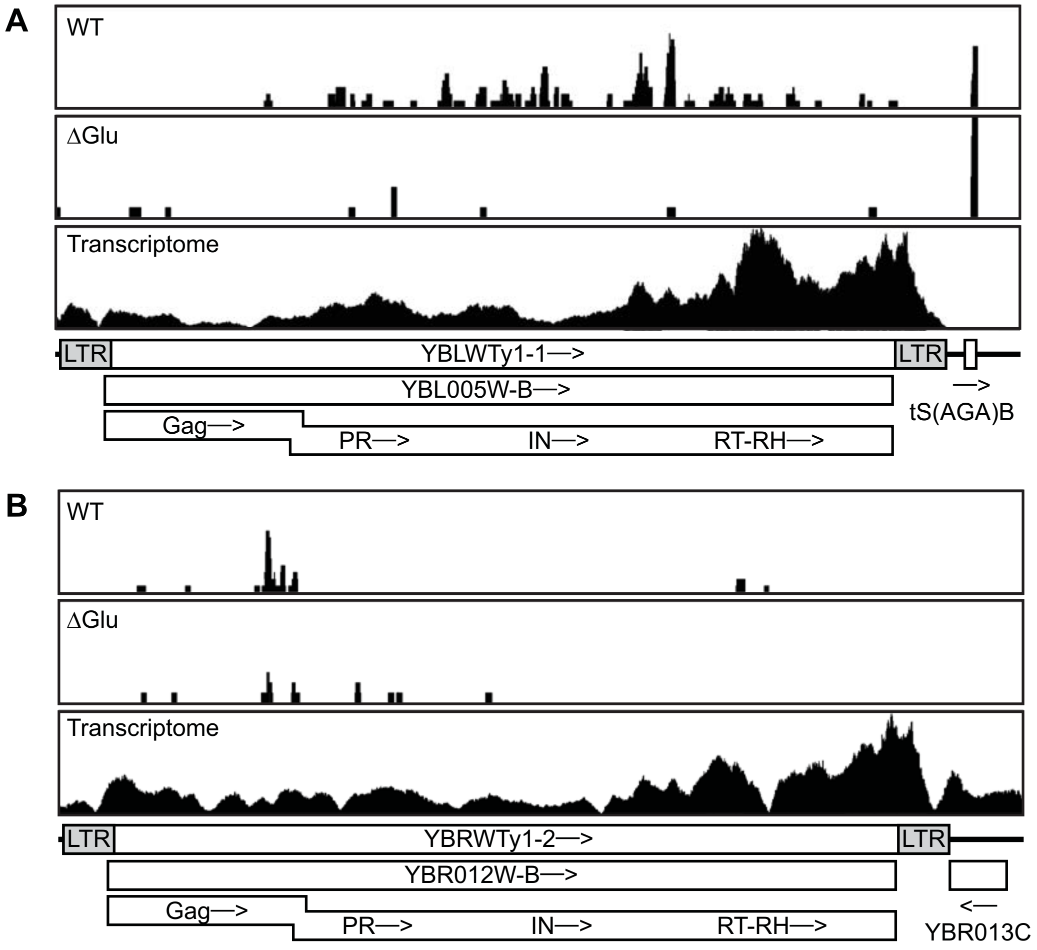

Supplementary Figure S9. Examples of crosslinks on retrotransposons. The Ty1–Ty4 retrotransposons express a 5' encoded encapsulation protein (Gag) and a fusion Gag-Pol protein that results from a +1 ribosome frameshift (reviewed by [11,12]). The long-terminal-repeat sequences (LTR) are shown in gray. The Pol ORF encodes an endonuclease (EN), an integrase (IN) and a reverse transcriptase-RNase H (RT-RH). The +1 frameshift occurs at a CUU-**A**GG-C, where the bold A is the position of the shift, that is within a 38-nucleotide overlap between the Gag and Pol ORFs. The shift from the CUU to UUA codon is enhanced by pausing of the ribosomes. **A**, Crosslinks on the Ty1-1 element YBL005W-B. which were primarily in the ORF of the Pol ORF. **B**, Crosslinks with the Gag ORF of the TY1-2 element YBR012W-A. The peaks map in purine-rich regions that are centered at ~250, ~150 and ~70 residues upstream of the frameshifted A. Data were analyzed with IGB and visualized with the IGV.

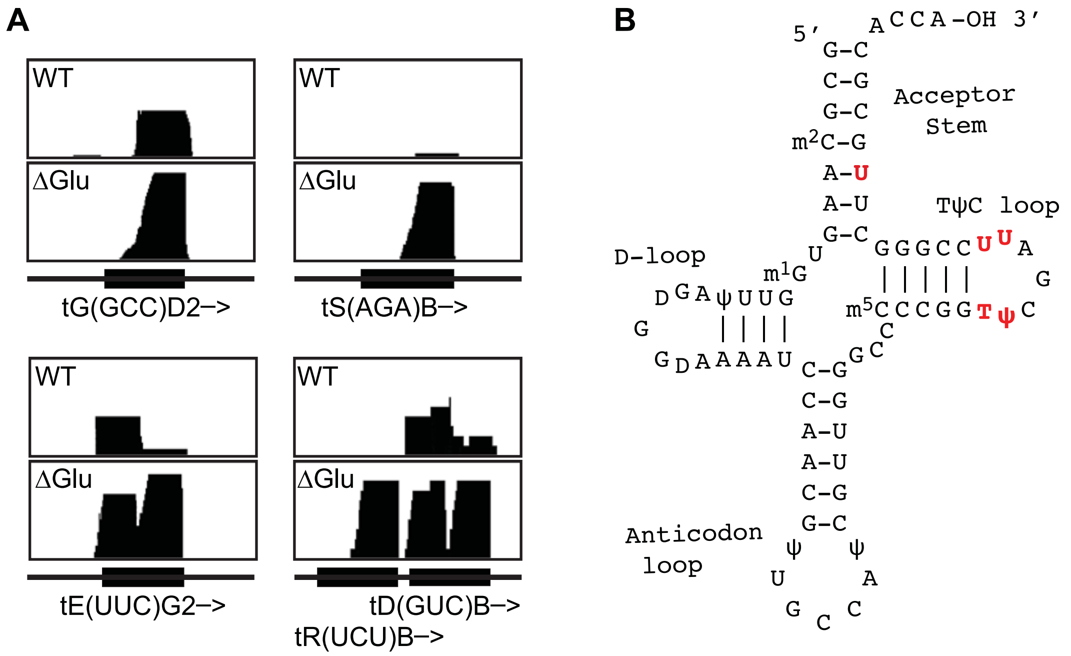

Supplementary Figure S10. Location of tRNA crosslinked fragments. Unique RNA fragments mapped on the indicated tRNAs. **A**, Examples of the IGV profiles of the tRNA fragments that generally mapped on the 3' half of the tRNAs. The orientation of the genes are as shown. Data were analyzed with IGB and visualized with the IGV. **B**, Positions of the U to C transitions mapped on tRNA-Gly are shown in red. These positions map to the elbow of the L-shaped three-dimensional structure.

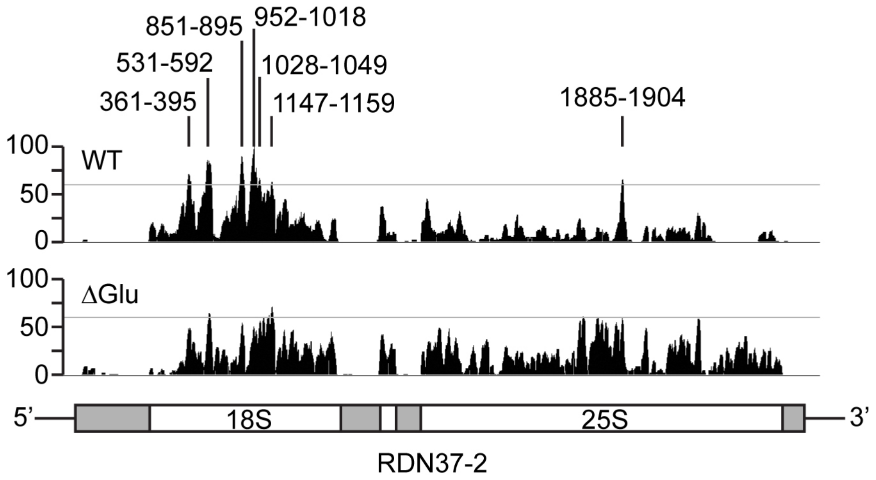

Supplementary Figure S11. Location of rRNA crosslinked fragments. Unique RNA fragments mapped on the 37S rRNA gene cluster using IGB and visualized with the IGV. The peaks with ≥ 60 reads are indicated with the cutoff shown as a light gray line. Helix 12 (H12) of the 18S is located at residues 361–382; H18 is at 557–588; and H24 is at 977–1027.

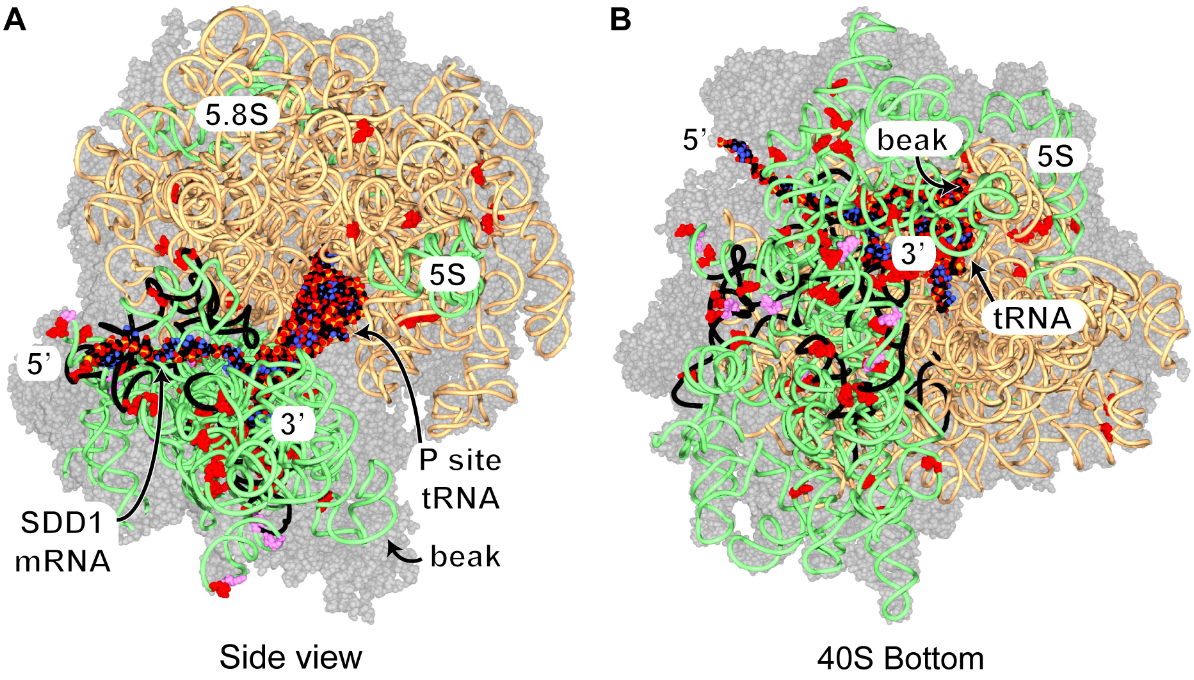

Supplementary Figure S12. The distribution of Ded1 crosslinks on rRNAs. The 40S and 60S ribosome proteins are shown with 50% transparency as gray space-filling models, the 18S, 5.8S and 5S rRNAs are shown as light green spaghetti, the 25S rRNA is beige spaghetti, and the bound tRNA and mRNA are shown as space-filling models. The positions of single or multiple U to C transitions are shown as red or magenta, respectively, space-filling models, and the RNA fragments highly crosslinked to Ded1 (≥ 60 reads) are shown as black. **A**, Side view along the translated SDD1 mRNA and showing the tRNA in the P site. **B**, View from the 40S bottom. The ribosome structure with bound SDD1 mRNA and tRNA in the P site (PDB 6snt) was used and viewed with Swiss PDBViewer [13,14].

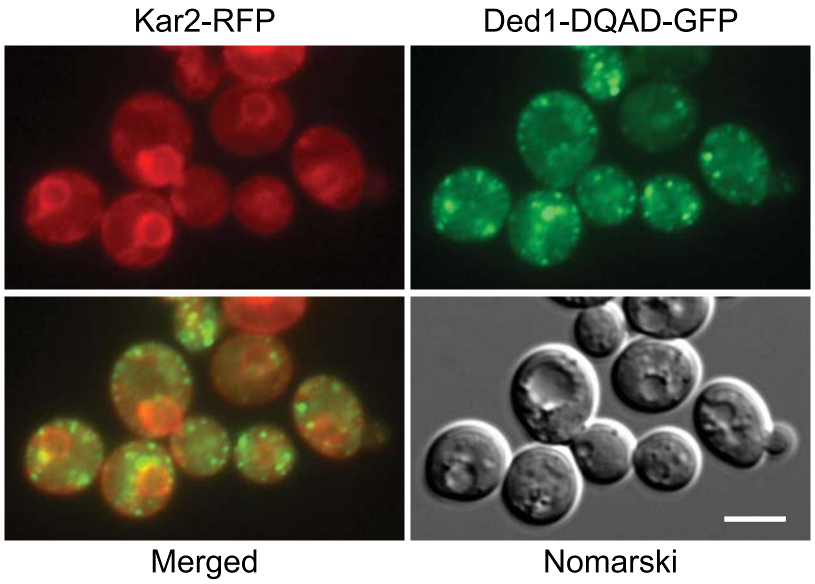

Supplementary Figure S13. Ded1-DQAD mutant forms foci adjacent to the ER. In yeast, the ER envelops the nucleus (central cisternal ER) and then extends as a cortical halo inside the plasma membrane of the cell wall (PM-associated ER) through interconnected tubules (tubular ER; [15]). Kar2 is a chaperone for refolding proteins within the ER lumen and thus is a marker of such [16]. Sec61 and Sec62 are part of the ER translocon pore needed for ER-associated translation; at a nonpermissive temperatures, the sec1 and sec62 temperature-sensitive (ts) mutants block protein import into the ER [17,18]. The ATPase-inactive Ded1-E307Q mutant (Ded1-DQAD) has a high propensity to form cellular foci with sequestered mRNAs that are no longer undergoing translation [19]. In the images shown, the sec62 ts mutant was incubated for 15 min at 37°C. The sec61 ts mutant gave similar results.

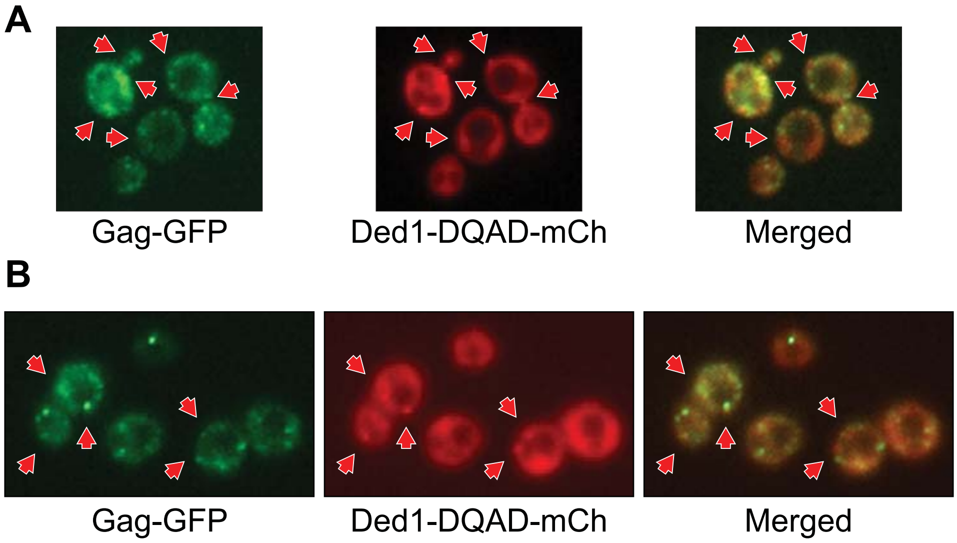

Supplementary Figure S14. Ded1 and Gag form foci that were in close proximity. Still images of selective frames of the movies Film Ded1-DQAD-mCh+gag-GFP and Film Ded1-DQAD-mCh+Gag-GFPΔGlu. Cells were grown to an OD600 of ~0.6 $ml^{-1}cm^{-1}$. The arrows indicate positions where the Ded1 and Gag foci were in close proximity. **A**, cells grown under standard conditions. **B**, cells grown under standard conditions were subsequently grown in the absence of glucose for 20 min.

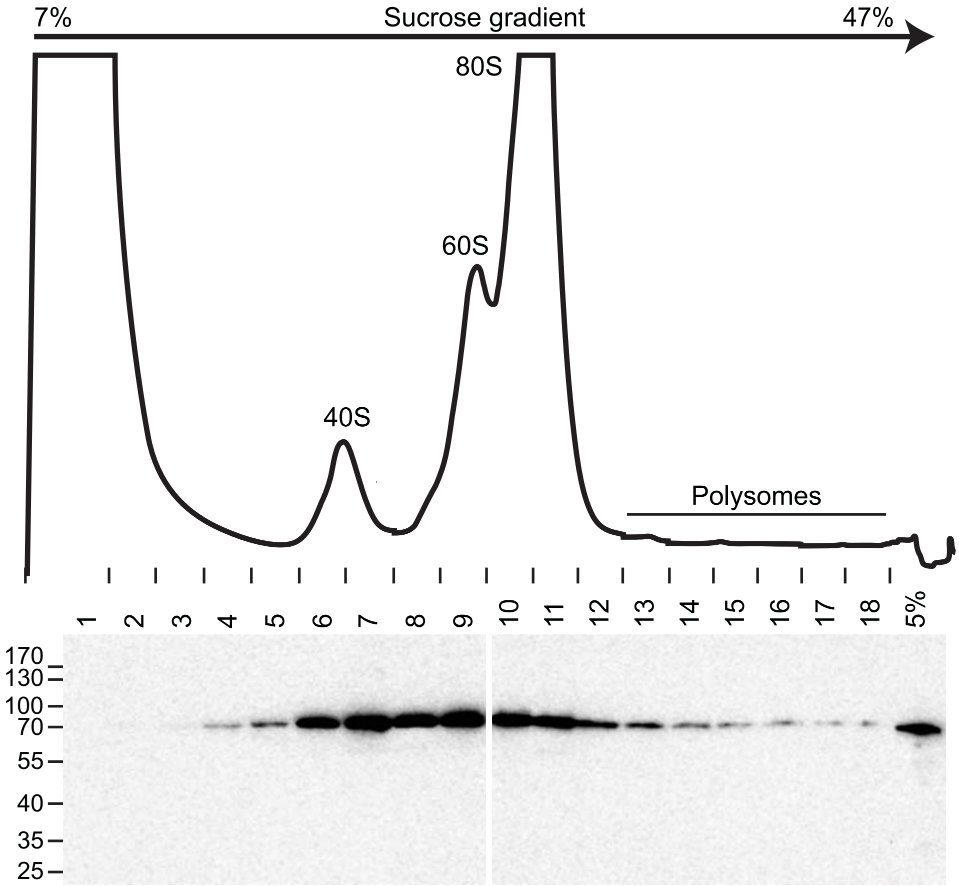

Supplementary Figure S15. The distribution of Ded1 after glucose depletion in a sucrose gradient. The cultures was incubated for ~5 min under glucose-depletion conditions before harvesting the cells. Cell extracts were incubated on ice with ~5 mM AMP-PNP and GMP-PNP just prior to loading on the 7–47% sucrose gradient. The Western blot of the SDS Laemmli gel shows the fractions containing Ded1. The last lane shows ~5% of the cell extract used for the gradient.

References

1. Horvath, A. and Riezman, H. (1994) Rapid protein extraction from *Saccharomyces cerevisiae*. *Yeast*, **10**, 1305-1310. 10.1002/yea.320101007
2. Hafner, M., Landthaler, M., Burger, L., Khorshid, M., Hausser, J., Berninger, P., Rothballer, A., Ascano, M., Jr., Jungkamp, A.C., Munschauer, M. *et al.* (2010) Transcriptome-wide identification of RNA-binding protein and microRNA target sites by PAR-CLIP. *Cell*, **141**, 129-141. 10.1016/j.cell.2010.03.009
3. Danan, C., Manickavel, S. and Hafner, M. (2022) PAR-CLIP: A Method for Transcriptome-Wide Identification of RNA Binding Protein Interaction Sites. *Methods Mol Biol*, **2404**, 167-188. 10.1007/978-1-0716-1851-6_9

4. Coleman, J.E. (1998) Zinc enzymes. *Curr Opin Chem Biol*, **2**, 222-234. 10.1016/s1367-5931(98)80064-1
5. Freese, N.H., Norris, D.C. and Loraine, A.E. (2016) Integrated genome browser: visual analytics platform for genomics. *Bioinformatics*, **32**, 2089-2095. 10.1093/bioinformatics/btw069
6. Khong, A., Matheny, T., Jain, S., Mitchell, S.F., Wheeler, J.R. and Parker, R. (2017) The Stress Granule Transcriptome Reveals Principles of mRNA Accumulation in Stress Granules. *Mol Cell*, **68**, 808-820.e805. 10.1016/j.molcel.2017.10.015
7. Lin, Z. and Li, W.H. (2012) Evolution of 5' untranslated region length and gene expression reprogramming in yeasts. *Mol Biol Evol*, **29**, 81-89. 10.1093/molbev/msr143
8. Pelechano, V., Wei, W. and Steinmetz, L.M. (2013) Extensive transcriptional heterogeneity revealed by isoform profiling. *Nature*, **497**, 127-131. 10.1038/nature12121
9. Bailey, T.L. and Elkan, C. (1994) Fitting a mixture model by expectation maximization to discover motifs in biopolymers. *Proc Int Conf Intell Syst Mol Biol*, **2**, 28-36.
10. Bailey, T.L., Johnson, J., Grant, C.E. and Noble, W.S. (2015) The MEME Suite. *Nucleic Acids Res*, **43**, W39-49. 10.1093/nar/gkv416
11. Curcio, M.J., Lutz, S. and Lesage, P. (2015) The Ty1 LTR-Retrotransposon of Budding Yeast, *Saccharomyces cerevisiae*. *Microbiol Spectr*, **3**, Mdna3-0053-2014. 10.1128/microbiolspec.MDNA3-0053-2014
12. Maxwell, P.H. (2020) Diverse transposable element landscapes in pathogenic and nonpathogenic yeast models: the value of a comparative perspective. *Mob DNA*, **11**, 16. 10.1186/s13100-020-00215-x
13. Matsuo, Y., Tesina, P., Nakajima, S., Mizuno, M., Endo, A., Buschauer, R., Cheng, J., Shounai, O., Ikeuchi, K., Saeki, Y. *et al.* (2020) RQT complex dissociates ribosomes collided on endogenous RQC substrate SDD1. *Nat Struct Mol Biol*, **27**, 323-332. 10.1038/s41594-020-0393-9
14. Guex, N. and Peitsch, M.C. (1997) SWISS-MODEL and the Swiss-PdbViewer: an environment for comparative protein modeling. *Electrophoresis*, **18**, 2714-2723. 10.1002/elps.1150181505

15. West, M., Zurek, N., Hoenger, A. and Voeltz, G.K. (2011) A 3D analysis of yeast ER structure reveals how ER domains are organized by membrane curvature. *J Cell Biol*, **193**, 333-346. 10.1083/jcb.201011039

16. Nishikawa, S.I., Fewell, S.W., Kato, Y., Brodsky, J.L. and Endo, T. (2001) Molecular chaperones in the yeast endoplasmic reticulum maintain the solubility of proteins for retrotranslocation and degradation. *J Cell Biol*, **153**, 1061-1070. 10.1083/jcb.153.5.1061

17. Deshaies, R.J. and Schekman, R. (1987) A yeast mutant defective at an early stage in import of secretory protein precursors into the endoplasmic reticulum. *J Cell Biol*, **105**, 633-645. 10.1083/jcb.105.2.633

18. Deshaies, R.J. and Schekman, R. (1989) *SEC62* encodes a putative membrane protein required for protein translocation into the yeast endoplasmic reticulum. *J Cell Biol*, **109**, 2653-2664. 10.1083/jcb.109.6.2653

19. Hondele, M., Sachdev, R., Heinrich, S., Wang, J., Vallotton, P., Fontoura, B.M.A. and Weis, K. (2019) DEAD-box ATPases are global regulators of phase-separated organelles. *Nature*, **573**, 144-148. 10.1038/s41586-019-1502-y
